# Insight into the hierarchical control governing leg stiffness during the stance phase of running

**DOI:** 10.1038/s41598-022-16263-7

**Published:** 2022-07-15

**Authors:** Alessandro Garofolini, Karen J. Mickle, Patrick McLaughlin, Simon B. Taylor

**Affiliations:** 1 Institute for Health and Sport (IHES), Victoria University, Melbourne, Australia; 2 School of Environmental and Life Sciences, University of Newcastle, Ourimbah, NSW Australia

**Keywords:** Control theory, Physiology

## Abstract

Leg stiffness plays a key role in the storage and release of elastic energy during stance. However, the extent to which a runner is able to reuse stored energy remains a limiting factor in determining their running effectiveness. In this study, ten habitual rearfoot strikers and ten habitual forefoot strikers were asked to run on a treadmill in three footwear conditions: traditional, neutral, and minimal running shoes. We examined the effect of habitual foot strike pattern and footwear on leg stiffness control within three task-relevant phases of stance (i.e. touch-down, loading, unloading). Control was quantified using stride-to-stride leg stiffness time-series and the coefficient of variability and detrended fluctuation analysis (DFA). The results are interpreted within a theoretical framework that blends dynamic systems theory and optimal feedback control. Results indicate that leg stiffness control is tightly regulated by an active control process during the loading period of stance. In contrast, the touch-down and unloading phases are driven mostly by passive allometric control mechanisms. The effect of footwear on leg stiffness control was inconclusive due to inconsistent trends across three shoe types. However, stiffness control was affected by landing technique. Habitual rearfoot strike runners have reduced DFA values during the touch-down and unloading phases. These sub-phases are associated with an allometric control process and suggests that rearfoot strike runners express a reduction in system complexity for leg stiffness control and hence, a less adaptable system.

## Introduction

For humans who engage in long distance running, the control of leg stiffness is important for performance and injury^1–3^. Experimental data and theoretical models from human and animal studies suggest that effective leg stiffness during steady-state running functions as to optimize the competing costs of energy, postural instability and injury risk^4–7^. Effective leg stiffness has been a useful expression of economy because it reflects global muscle–tendon unit behaviour^8^. For example, energy efficient solutions are achieved when sufficient energy in the muscle–tendon unit is stored and recovered from the loading phase to the unloading—propulsive—phase^9–12^. Clinical studies of human running support an ideal leg stiffness range for safe locomotion, with evidence that suggests skeletal injuries are associated with too-high leg stiffness, while muscle–tendon injuries are associated with too-low leg stiffness^13,14^. Hence, a runner’s locomotor system must control leg stiffness to optimise task priorities. However, the nature of leg stiffness control, and the neuro-mechanical factors that influence control, are mostly unknown. Understanding the process for leg stiffness control both within a period of stance and between cycles, will provide valuable insights for multiple issues surrounding running performance, musculoskeletal loading of body structures and injury prevention.

Motor solutions that utilise elastic structures to store and contribute energy are essential for efficient and effective propulsion^12,15^. Muscle–tendon units store this energy during the loading phase and this requires control of a unit’s force–length dynamics^16^. This control task is made challenging when external forces are unpredictable, such as foot-to-ground contacts during running. Approximately 80% of experienced runners use impact-assistive shoes and adopt a rearfoot strike (RFS) loading technique during touchdown^17–19^. When such loading patterns are compared with a minimal-assisted forefoot alternative, the RFS pattern has a larger leg effective mass^20,21^ and inconsistent, high-frequency, force loading profiles^20,22–24^. Hence, it is reasoned that the landing technique is likely to affect the muscle–tendon unit mechanism for the purpose of energy storage during loading and, the subsequent utility of energy recovery during unloading. Given that optimising leg stiffness is a priority of running, and stiffness control is affected by condition of landing technique and substrate compliance, then a change to any combination of these two conditions should be matched with an active goal-relevant response by the locomotor controller.

There is contrasting evidence on the effect of foot strike and footwear on leg stiffness, with some reporting greater impact loading and a stiffer leg during landing when wearing more cushioned shoes^25–27^; while others found no differences between running in traditional and maximalist footwear and also a more rigid limb in minimalist footwear^28^. Discrepancies in these findings may reflect an altered motor control strategy to adjust leg stiffness during the time course of loading and unloading. Before we propose our predictions and a suitable test method, a brief overview of human motor control theory is necessary to setup our paradigm of leg stiffness control.

**Figure 1:**
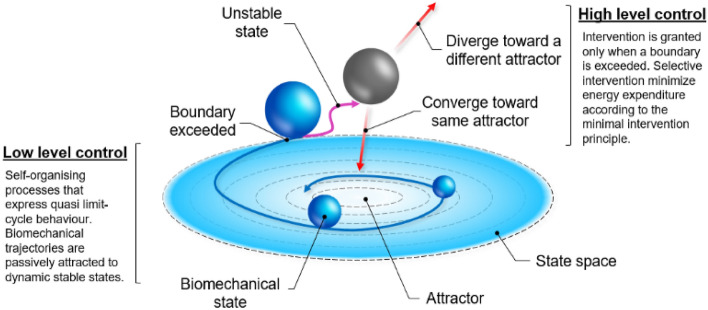
Theoretical hierarchical control model. According with the dynamical systems theory and optimal feedback control theory, motor actions are controlled by a two-level hierarchical structure. The low-level controller is a self-organising entity exploiting the inherent complexity of the system; its allometric control allows solutions to emerge and flexibility to increase. The high-level controller actively supervises motor action and intervenes only when necessary. This minimize the costs associated with active intervention such as excessive energy expenditure, less variable performance, and unrestricted workspace. This figure has been generated by researcher AG using Windows PowerPoint 2016.

The locomotor control system is often considered as a hierarchical structure, and accordingly, it has been successfully modelled by combining two relevant theories^29,30^. Dynamical systems theory^31^ and optimal feedback control theory^32^ represent the levels of this hierarchical structure (Fig. 1). The system is actively supervised by a high-level controller that adheres to the principle of minimum intervention^32–34^. Because intervention is costly, the controller prefers to manage task goals by deferring to a low-level self-organising processes that expresses quasi limit-cycle behaviour, where biomechanical trajectories are passively attracted to dynamic stable states^35,36^. Optimal control also becomes easier when part of the problem is offloaded to the low-level controller^30^. In essence, the locomotor control system will express complex self-organisation behaviour, unless intervention is warranted by a higher-level supervisor. A variety of evidence shows that the structure of complex limit-cycle behaviour is modified when this ‘lower-order’ system is subjected to an external ‘higher-order’ intervention^33,37,38^. The ‘lower-order’ property of the model belonging to complexity and dynamical systems theory has relevance for the question in this study: how does shoe-assisted rearfoot loading influence the inherent flexibility of the locomotor control system that is relevant to leg stiffness? The premise is that a high degree of system complexity (rich dimensionality of system resources) is desirable for the high-level controller that prefers minimal regulation of control. Therefore, by quantifying complexity we can observe relative level of control regulation, and hence we can gain insight of the embodied ‘low-order’ state of the locomotor system that is relevant to the default mode of stiffness control.

Among many tools that quantify system complexity, one approach—Detrended Fluctuation Analysis (DFA)—has successfully demonstrated an ability to detect the level of intervention by the central nervous system to regulate locomotor control by examining persistence (i.e. a scale of self-similar structure) in the time-series of a known control parameter or performance variable of gait^37,39–41^. Gait parameters that express time-series persistence are considered to be an expression of a complex self-organised system^37,42,43^, while anti-persistent structure (random correlations) suggests higher-order intervention^33,44^. Therefore, if the high-level controller top-down intervention is active then it will cause an interruption to time-series persistence (i.e. low DFA value), and a subsequent sensitive change to the task performance (i.e. high coefficient of variation) will be evident because the high-level controller would only choose the cost of intervention if it could effect a sufficient change to a task goal. Also, within an embodied system there will be no change to the complexity of the system provided that the conditions of the task are consistent.

If we can discern when the higher-levels of the locomotor control system intervene to regulate leg stiffness, then we can gain important insights into human running that extend beyond issues of foot strike technique and footwear design. The interplay of a hierarchical control system is a critical issue that is overlooked in nearly all studies that use an analysis of time-series persistence to gain insight into the human locomotor system, where many studies are limited to interpret their findings through a restricted framework (for example^45,46^). With the exception of Dingwell, Salinas^47^, there are no studies (investigations of the gait control system) that have attempted to reconcile the quantification of dynamic fluctuations of critical (goal-relevant) parameters through the framework of a two-level hierarchical control system^48^. This study is the first to afford an insight into the dual nature of the interactive control systems governing the determinants of running, specifically leg stiffness.

We test the hypothesis that two different types of runners classified by foot strike landing technique with habituation to a specific substrate (i.e. footwear assistance), will show differences in their regulation of leg stiffness control. We expect this difference to be due to their landing technique and their embodied neuro-musculoskeletal systems they acquire from this habitual condition. In addition, we test the hypothesis that when the runners perform a repeat test using the same landing technique, but receive a change in the level of footwear (substrate) assistance, we expect to see a relative change to their control regulation and a proportionate relative change to the consistency of their task performance. The outcomes will form support for the theories of leg stiffness control during running and add important new insights to complement the debate about the effect of footwear and landing technique on human running, which up till now has been dominated by biomechanics perspective. Furthermore, the results will provide evidence for the existence of a hierarchical locomotor control system that defaults to a ‘low-level’ embodied with complexity (i.e. flexibility).

## Results

### Leg stiffness control policy during the stance phase

The investigation of control responsibility confirmed that both groups adopt the same k_leg_ control policy: high-level intervention control occurs at K2, while low-level control occurs at K1 and K3. This claim is based on the correlations of ΔDFAα with ΔCV, which were consistent for both RFS and FFS (Table 1). There was a significant main effect of *Phase* on DFAα (p < 0.001, Table 2), indicating that DFAα was dependent on stance phase task. Post hoc tests reveal that DFAα is higher (p < 0.001) at K3, compared to K2. The variability of the final force–length state at the end of the loading phase (ΔEllipse Area) was correlated with both ΔDFAα and ΔCV (Table 3) showing that reduced ΔDFAα is associated with increased ΔEllipse area.

**Table 1:** Pearson correlation coefficient *r* between ΔDFAα and ΔCV for each task-dependent phase of stance (K1, K2, and K3).

	Correlation	Group		All
		FFS	RFS	
ΔCV	ΔCV_K1_ − ΔDFA_K1_	0.2 (p = 0.503)	0.2 (p = 0.377)	0.2 (p = 0.285)
	ΔCV_K2_ − ΔDFA_K2_	− 0.6 (p = 0.004)	− 0.7 (p < 0.001)	− 0.6 (p < 0.001)
	ΔCV_K3_ − ΔDFA_K3_	0.4 (p = 0.111)	0.1 (p = 0.605)	0.1 (p = 0.515)

Results were also combined for RFS and FFS (All).

**Table 2:** Primary statistical results for differences between groups, shoes, and region for mean leg stiffness, coefficient of variation (CV), and mean alpha values.

Variable	Group	Shoe	Phase	Group × shoe	Group × phase	Shoe × phase	Group × shoe × phase
Mean	**F**_**(1,38)**_** = 5.35 p = 0.026**	**F**_**(2,76)**_** = 17.55 p < 0.001**	**F**_**(2,76)**_** = 176.94 p < 0.001**	**F**_**(2,76)**_** = 10.83 p < 0.001**	**F**_**(2,76)**_** = 4.42 p = 0.041**	**F**_**(4,152)**_** = 19.33 p < 0.001**	**F**_**(4,152)**_** = 11.94 p < 0.001**
CV	**F**_**(1,38)**_** = 5.31 p = 0.027**	**F**_**(2,76)**_** = 12.97 p < 0.001**	**F**_**(2,76)**_** = 90.06 p < 0.001**	**F**_**(2,76)**_** = 8.03 p = 0.003**	**F**_**(2,76)**_** = 4.79 p = 0.031**	**F**_**(4,152)**_** = 5.66 p = 0.007**	F_(4,152)_ = 0.54 p = 0.561
Alpha	**F**_**(1,38)**_** = 5.31 p = 0.027**	F_(2,76)_ = 1.42 p = 0.250	**F**_**(2,76)**_** = 14.69 p < 0.001**	F_(2,76)_ = 0.06 p = 0.942	F_(2,76)_ = 2.25 p = 0.113	F_(4,152)_ = 0.35 p = 0.846	F_(4,152)_ = 1.60 p = 0.178

ANOVA results are given for main effects and interactions.

Statistically significant findings are in bold.

**Table 3:** Pearson correlation coefficient *r* between ΔEllipse area and ΔDFAα and ΔCV for RFS and FFS.

	ΔDFA		ΔCV	
	FFS	RFS	FFS	RFS
ΔEllipse area [norm]	− 0.2 (p = 0.536)	− 0.6 (p = 0.074)	0.8 (p = 0.021)	0.7 (p = 0.055)

For FFS, changes in ΔEllipse area, DFAα and CV were computed between running trial in moderately assisted shoe (med MI) and in minimal assisted shoe (high MI). For RFS changes were computed between running trial in high assistance shoe (low MI) and in moderate assisted shoe (med MI).

### Group effect on the control of leg stiffness

**Figure 2:**
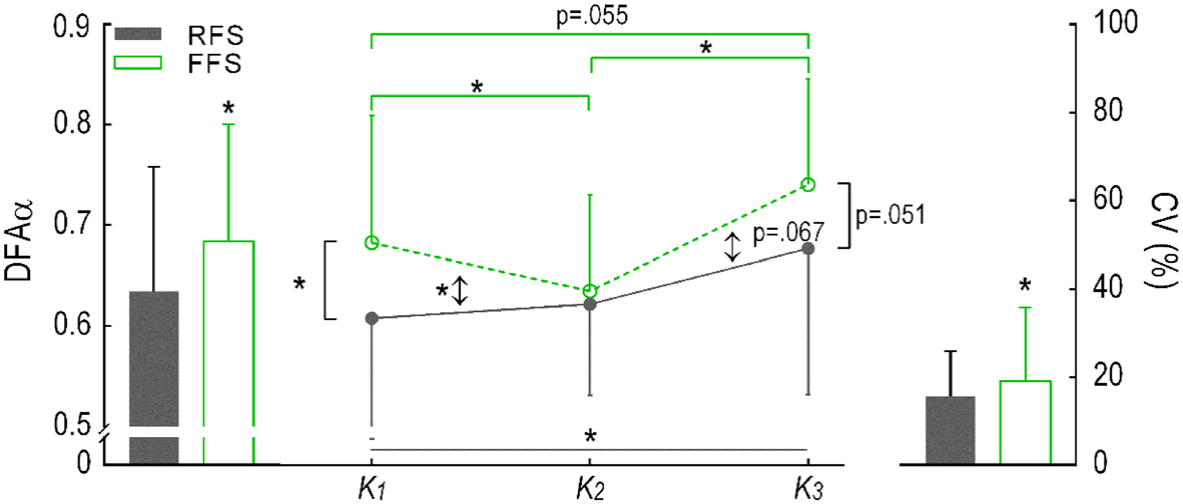
Group mean and SD of DFAα values averaged across shoe types for each group, and over the three task-relevant sub-phases of the stance phase. Bar graphs show between-group (FFS vs RFS) differences for average DFAα and average CV across sub-phases and shoe type. *Represents significance level p < 0.05; for *group* × *phase* interaction effects, and pairwise comparisons for between group and between phase.

**Figure 3:**
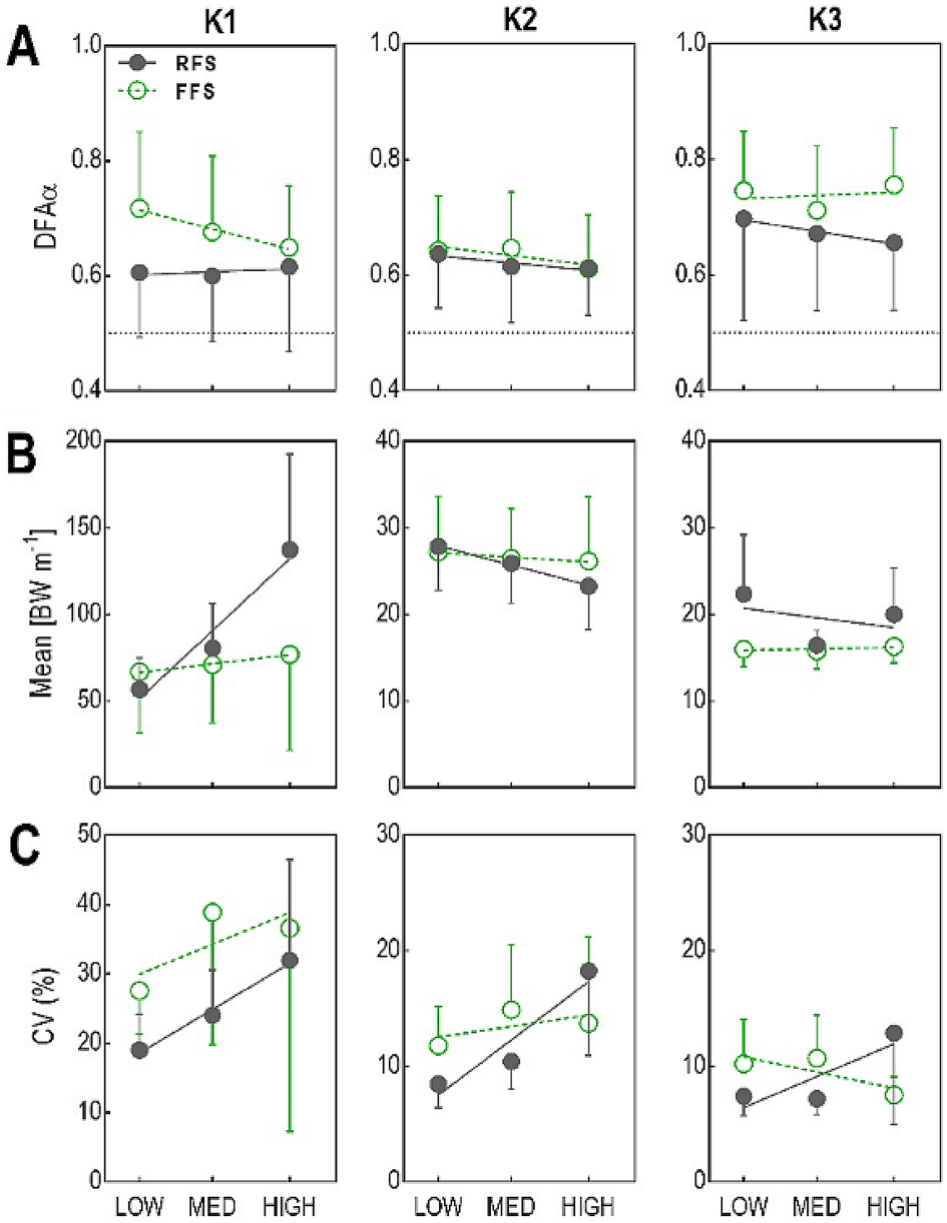
Group mean and SD for each task-relevant phase (K1-K3) and shoe type (LOW, MED, HIGH) for dependent variables: (**A**) DFAα of leg stiffness, (**B**) mean leg stiffness, and (**C**) CV of leg stiffness. Magnified scale in (**B,C**) for sub-phases K2 and K3.

There was a higher DFAα for FFS compared to RFS indicated by a significant main effect for *Group* (p = 0.027, Table 2). There was a trend for a *group* × *phase* interaction effect on the DFAα (p = 0.113, Table 2, Fig. 2), indicating a potential difference between groups in the way they regulate the control of stiffness between phases. Planned contrasts compared the groups change in DFAα between phases K1 and K2 (p = 0.017) and between K2 and K3 (p = 0.067). Groups differ in their control policy from touch-down phase (K1) to loading phase (K2), while both groups reduce tight control of k_leg_ during transition from loading (K2) to unloading (K3); in this transition, the FFS group made a relatively higher change to DFAα compared to RFS (Fig. 3). Pairwise comparisons within-FFS group show they have higher DFAα at both touch-down (p = 0.044) and unloading (p < 0.001) when compared to loading.

### Interaction effect of phase and group on leg stiffness control

Figure 3 demonstrates the effect that the experimental conditions had on mean k_leg_ (3B) and its variability (3C), and how this differs between group, and phases. However, there are no consistent trends between k_leg_ variance (3C) and control regulation (3A) when examining group behaviour. For the dependent variables CV and mean k_leg_ the significant main effect of *Phase* (p < 0.001; Table 2, Fig. 3) was not unexpected. Pairwise comparisons show that k_leg_ is stronger (p < 0.001) and more inconsistent (p < 0.001) at K1 compared to K2; while k_leg_ at K2 is stronger (p < 0.001) and more inconsistent (p < 0.001) compared to K3 (Supplementary Appendix Table S1). However, while both groups display a similar mean (p > 0.05) and CV (p > 0.05) of k_leg_ during K2, their behaviour at K1 and K3 is different (Supplementary Appendix Table S1). Hence, there was a significant interaction effect of *Phase* × *Group* on mean and CV of k_leg_ (p = 0.041 and p = 0.031 respectively; Table 2, Fig. 3). For direct within-group pairwise comparisons between K1 and K3, both groups had a greater (p < 0.001) mean k_leg_ and a larger (p < 0.001) CV at the touchdown phase (K1). For between-group comparisons, RFS exhibited a greater (p = 0.034) mean k_leg_, while FFS exhibiting larger (p = 0.023) CV.

### Shoe effect on control of leg stiffness

For the dependent variable DFAα: shoe did not have a significant effect on the interaction between *group* × *phase* × *shoe* (p = 0.178; Table 2, Fig. 3); there was no main effect for *shoe* (p = 0.250), nor interaction effects for *shoe* × *group* (p = 0.942) or *shoe* × *phase* (p = 0.846). Therefore, the interaction effect of *shoe* did not change the *group* × *phase* behaviour identified in our second hypothesis.

## Discussion

We investigated three hypotheses related to the control of leg stiffness in human running. First, that leg stiffness is governed by tight control regulation during the loading phase. Second, that leg stiffness control will be affected by landing technique (rearfoot, forefoot). Third, that footwear assistance will reduce the need for control regulation of leg stiffness. In summary, we found that the control system responsible for k_leg_ varies between high-level and low-level control processes, and this dual interplay depends on the task objective inherent to the stance sub-phases: touch-down, loading, and unloading. Second, we found that control processes that regulate k_leg_ are affected by the foot strike technique adopted. Third, while mean k_leg_ of RFS runners was dependent on footwear assistance and contrasted with forefoot strike runners whom maintained consistent mean k_leg_ irrespective of footwear assistance, the acute effect of footwear assistance on control capacity of k_leg_ was not conclusive. These hypotheses are addressed in detail below.

The first hypothesis was supported, where control regulation of k_leg_ shows variable contributions of high- and low-level control systems, and is task (sub-phase) dependent. While a low DFAα during the loading phase suggests high-level intervention, if the associated effect is unknown then this result could simply be an expression of reduced complexity inherent to low-level control processes. We overcame this dilemma by analysing a recognised control parameter of running (k_leg_), and by subtle variations of experimental conditions (i.e. footwear type) that probe k_leg_ control. Hence, we were able to associate a change in control process (statistical persistence) of k_leg_ with change-in performance (magnitude of the variance) of k_leg_, and therefore reconcile the responsible system that regulates k_leg_. The significant negative correlation between k_leg_ persistence (DFAα) and k_leg_ variability (CV) during the loading phase supports the claim that an increase in k_leg_ CV is associated with decreases in DFAα. A similar association between variance magnitude of performance and underlying control process from the subtle probing of gait conditions has been recently supported^39^. This negative correlation is expected because undesirable states of k_leg_ should require regulation and active intervention from high-level control. Because there are varied initial states of k_leg_, there will be an equally varied response in k_leg_ to return the leg force–length dynamics to the goal-state. In other words, when the system makes a large response by producing a highly consistent k_leg_ from highly variable initial state, we find that these cases are associated with low DFA values. This reduction in DFA is most likely the expression of high-level control intervention.

**Figure 4:**
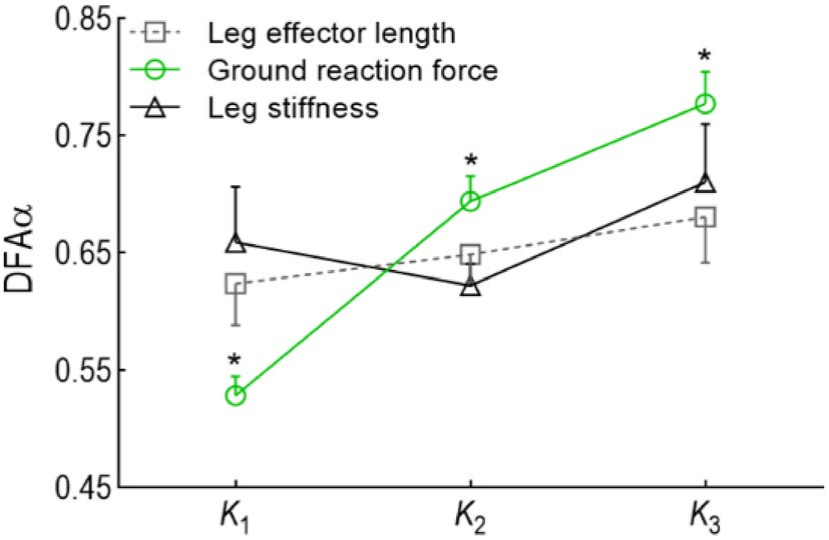
Mean and SD of DFAα values for leg length effector, ground reaction force, and leg stiffness averaged across shoe types and group, over the three task-relevant sub-phases of the stance phase. *Represents significance level p < 0.05; for component × phase interaction effects, and pairwise comparisons for between component within phase.

To support our hypothesis that k_leg_ is indeed the controlled variable during the loading phase (K2), we also tested the persistency during the sub-phases of the stance of the two components (change in force, and change in leg length) that we used to compute k_leg_. One could argue that control of leg stiffness could be achieved indirectly by direct control over one of the two components. We found that DFAα values for leg stiffness were lower during the loading phase (K2) than either change in force or change in leg length (Fig. 4) confirming direct control over leg stiffness.

During touch-down (K1) the controller works to stabilize external forces exploiting the flexibility in leg length and leg stiffness. While during unloading (K3) the controller is more attentive to changes in leg length component than the force. Over the stance phase the priorities of the control system change so that safety is prioritized at touch down (by controlling external forces), storing of elastic energy is prioritized during loading (by controlling leg stiffness), and stability of body centre of mass trajectory is prioritized during unloading (by controlling leg length).

Our expectation that k_leg_ during the unloading phase would be governed by low-level control process was supported. The unloading of the leg is where stored elastic energy of the muscle–tendon unit is recovered from the eccentric loading phase to assist with leg extension and forwards acceleration of the body^15^. The result that FFS have lower k_leg_ during unloading (Fig. 3) suggests that for FFS runners, their leg lengthening can be achieved with less force contribution. This could be due to their body posture (ease of lengthening for a given GRF), but it could be due to passive contributions from tendon utility. The relatively high persistence (DFA) of k_leg_ during unloading indicates the high-level controller defers to the low-level controller (i.e. minimal intervention). This affords some advantages for optimal control of k_leg_. First, by avoiding high-level intervention the search for movement solutions through the neuro-motor workspace can occur relatively unrestricted^49^. In theory, this workspace expansion enables a more efficient allometric search process to find the coordinated motor commands that optimise energy recovery from the given initial state expressed by the muscle–tendon units. Second, allometric control is associated with less variability in performance (Table S1). Third, allometric control minimises the energy expense caused by decision making by the high-level controller^50^. This default strategy of deferring to the low-level controller (also referred to as the minimal intervention principle) is based on theoretical construction behind successful simulation studies that follow an efficient search process to achieve optimal control^30,49,51^.

Based on the premise of low-level control being the default mode, we also expected k_leg_ control during the touch-down phase to be under low-level control because it is a brief transitory period, which would be difficult for high-level control processing to achieve a target outcome^52^. Support for this hypothesis was evident in the FFS group based on two criteria: first, there was a relatively high DFA compared to loading phase, and second, any change in DFA was not associated with a change in CV. In contrast, while the RFS group demonstrated no association of the ΔDFA-ΔCV relationship, they also did not demonstrate a relatively higher DFA during touch-down. There are two explanations for a low DFA during touch-down: first, the complexity of the system is reduced; and second, there is high-level control during the pre-landing phase. The first explanation is most likely because we found no association of ΔDFA-ΔCV for the RFS group (i.e. no beneficial effect on k_leg_ consistency if there was investment of high-level control). In all cases, the k_leg_ variance was highest during the primary touch-down phase (i.e. an inconsistent k_leg_ state); thus to compensate it is reasonable to expect tight regulation by the high-level controller to achieve a given goal-state at the conclusion of the loading phase. In contrast, because DFA change was not correlated with k_leg_ change during the touch-down and unloading phases, we reason that inherent allometric control is responsible for governing leg force–length dynamics during these sub-phases of stance.

Based on the criteria above, we conclude that long-distance runners with a rearfoot strike landing technique express a reduction in system complexity for controlling k_leg_, therefore confirming our second hypothesis. Results suggest that RFS running may enhance specialisation of biomechanical patterns (low CV) but at the expense of flexible force–length solutions; RFS runners have a more consistent k_leg_ (i.e. low CV) during both touch-down and unloading phases.

We expected that runners would require less control regulation when running in their preferred shoe type compared to less-preferred shoes. Based on the knowledge that even small changes in habitual shoes’ properties can influence the maintenance of leg stiffness^53^, alter performance^54^ and could deeply modify the intricate muscle–tendon mechanics of running^55^, leg stiffness control should also increase away from habitual conditions. However, we found that during touch-down phase (K1) of FFS there was a tendency for system entropy to reduce when the shoe assistance was reduced (Fig. 3A, Table 2); this was unexpected because reduced footwear assistance (high MI index) was expected to expand system dimensionality and expression of complexity^56,57^. Nevertheless, compared to RFS that kept a constant level of DFAα, FFS adapted the control of k_leg_ as they changed shoe. The effect of habitual RFS running seems to affect the embodied complexity of their inherent locomotor search space for regulating leg stiffness.

With this knowledge, runners, coaches, and clinicians may select a combination of foot strike and footwear conditions that challenge k_leg_ control at specific stance sub-phases on the basis of training (performance) and/or rehabilitation goals. Based on the finding that the landing is less critical for FFS, because they can defer to the allometric control, and that FFS lower k_leg_ during unloading suggests a better ‘bounce’, or energy return, we also speculate that forefoot strike technique gait retraining may be recommended for safety and efficiency^25,58^.

**Figure 5:**
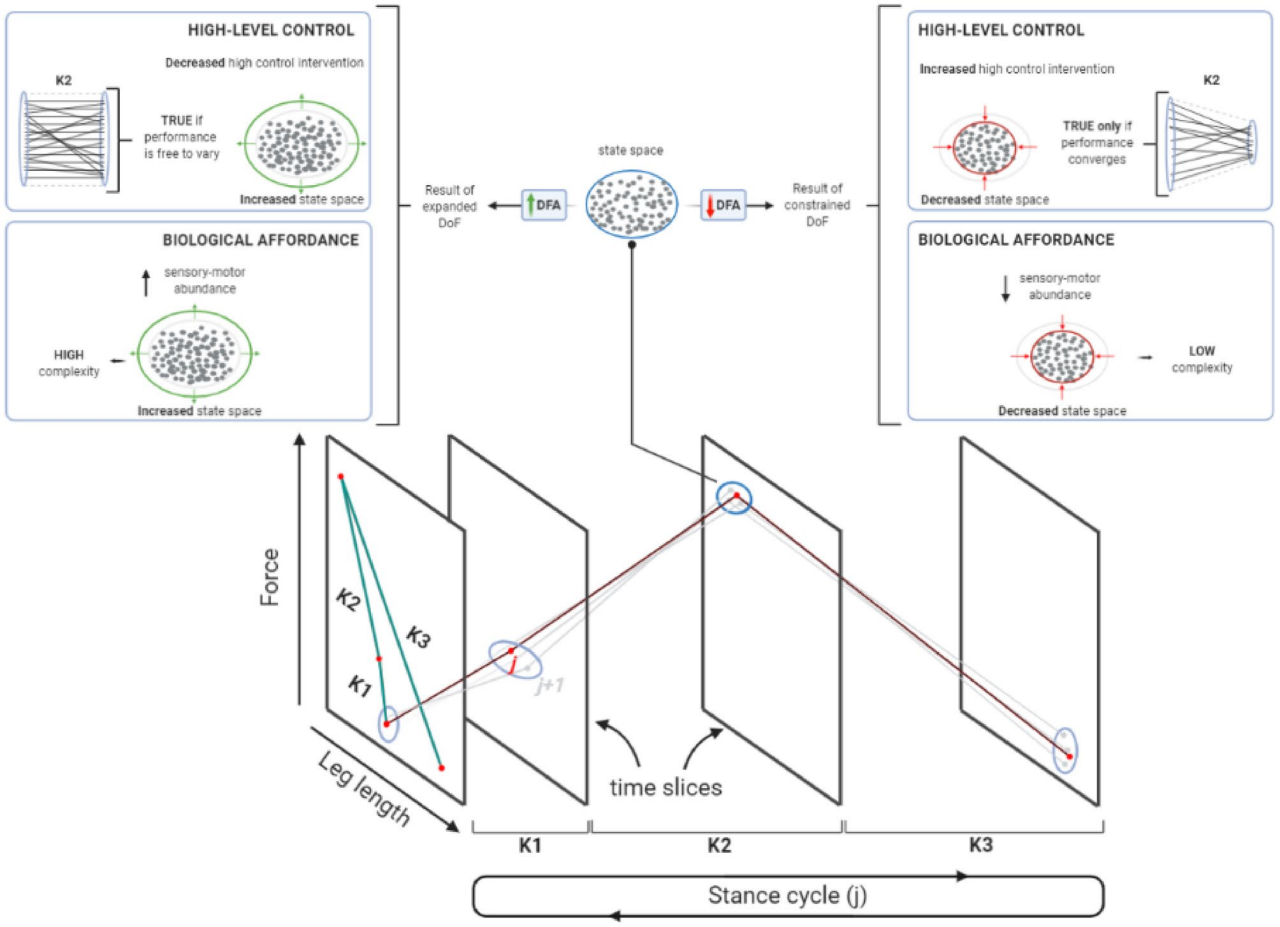
Theoretical framework to interpret DFA results of leg stiffness. Leg stiffness is computed within three sub-phases (K1–3), based on the respective change in the initial and final leg force–length states. The persistence of leg stiffness is quantified from a time-series of multiple stride cycles (j, j + 1,…, j + n), forming a distribution of slope values. The DFAα value is determined by factors that either restrict or expand the degrees of freedom (DoF) of the embodied neuro-motor system. This modulation is due to the combined interaction of two general factors: high-level control and inherent biological affordance. For example, if the change in length–force (i.e. between K1 and K2) is free to vary the system can express its full complexity resulting in a high DFA α value. On the other hand, if there is an increased high control intervention or an inherent low complexity of the system the DFA α value is low (right panel). In this case, if the change in length–force profile is constrained toward a smaller portion of the state space it represents the active high control intervention; if not, the system expresses its low complexity. This figure has been generated by researcher AG using Windows PowerPoint 2016.

This study has several limitations. First, we considered that shoes classified by a minimalist index ‘MI’ provides equivalent loading and unloading control assistance for both RFS and FFS runners. It is possible that assistance can change between loading and unloading. Furthermore, a low MI shoe could be assistive for a RFS runner but un-assistive for a FFS runner. The different effects of shoe on group could have prevented the identification of optimal shoe-type for optimal loading–unloading control. Second, we interpret DFAα results as representing the compound effects of system complexity (expansion of degrees of freedom dimensionality) and active top-down control regulation (Fig. 5). We based our interpretation on the idea that the active top-down control intervention is expressed by a relative change in two properties (a decreased DFAα and increased CV), meaning that an active top-down control intervention must exist because k_leg_ converges towards a less variant state. Future studies should test this theory by validating appropriate model simulations of inter-related parameters (e.g. leg force–length and leg stiffness) with empirical data (e.g.^33^). Last, we have to acknowledge a limited sample size and a gender restriction that limits generalisation of the results. The strict inclusion criteria were necessary to ensure the sample of selected runners was an appropriate representation of the population they were intended to represent and their demographics were equivalent between groups (i.e. body mass, average running load per week).

To conclude, we provided evidence for the dual nature of the interactive hierarchical control systems governing leg stiffness during running, and we showed how the task specific sub-phase of loading required a greater contribution of higher-level control intervention while the landing and unloading sub-phases defer governance to self-regulatory lower-level control. The likely reason for high-level intervention during loading can be attributed to a combination of competing and compounding cost factors that cannot be optimized simultaneously (e.g. energy, stability, and injury avoidance). While explicit nature of control policies was outside the scope of this study and require future investigation, the essential effect was consistent across all runners. However, habitual rear foot strike runners may have developed a restricted repertoire of biomechanical patterns at the expense of flexible force–length solutions to regulate leg stiffness. While habitual forefoot strike runners may rely on such flexibility to make landing safer and unloading more efficient.

## Methods

### Participants

A priori power calculation was conducted with the program G*POWER^59^; based on previous studies^60,61^ ten participants for each group were required to detect an effect size of 0.3 with 80% power and a 5% significance level using repeated measure, within-between interactions (ANOVA). Twenty competitive male long-distance runners (age: 31.2 ± 6.9 years, height: 1.77 ± 0.07 m, weight: 73.4 ± 7.9 kg, training load: 83 ± 22.5 km/week, age-graded score: 67.8 ± 6.4%) met the following inclusion criteria: running volume of at least 40 km/week, had been free of neurological, cardiovascular, and musculoskeletal problems (i.e. pain, soreness or injuries) within the previous six months. The age-graded score was computed via (www.howardgrubb.co.uk/athletics/wmalookup06.html) according to runners age, gender, and self-reported best race performance, similar to^62^. All participants were classified as competitive runners given an age-graded score of > 60%^63^. Participants gave their written informed consent to be part of the study. They were grouped according to a foot strike classification criteria proposed by Garofolini, Taylor^64^: forefoot strike runners (FFS, n = 10) if they demonstrated a higher proportion of ankle plantar-flexor work during the initial touch-down period of stance; conversely, rearfoot strike runners (RFS, n = 10) had a larger proportion of ankle dorsi-flexor work. The Victoria University Research Ethics Committee has approved the study (No. HRE16-061). All research was performed in accordance with the Declaration of Helsinki.

### Experimental protocol

Running tests were performed on an instrumented treadmill (Advanced Mechanical Technology Inc., Watertown, MA, USA) collecting three-dimensional ground reaction force data at 1000 Hz. Our instrumented treadmill included a stiffening frame to reduce the effect of low resonant frequencies^65^. Three-dimensional kinematics data were collected at 250 Hz by a fourteen cameras VICON system (Oxford Metrics Ltd., UK), and time-synchronized with ground reaction force data within the same system. A trunk and lower-limb biomechanical model was reconstructed from 45 retroreflective markers, for model details see Garofolini, Taylor^66^.

After completing a standardized and progressive 7-min warm-up, participants repeated a 5-min running test three times, with a different shoe for each trial. The three shoe models were differentiated by their minimalist index (MI); a range from 0 (maximum assistance) to 100% (least interaction with the foot)^67^. The shoes adopted in our experiment were classified at low MI (Mizuno Wave Rider 21, MI = 18%), medium MI (Mizuno Wave Sonic, MI = 56%), and high MI (Vibram Five Fingers, MI = 96%) see Supplementary Appendix Table S2. Treadmill speed was fixed at 11 km/h for all test conditions as this was a comfortable pace for all participants.

### Data analysis

Raw kinematic and kinetic data were exported from Nexus 2.6 (VICON, Motion Systems, Ltd., Oxford, UK) to Visual 3D (C-motion Pty, USA) for processing and parameterisation. The kinematic and kinetic signals were low-pass filtered using a 4th order, zero lag, Butterworth filter, with cut-off frequencies of 15 Hz and 35 Hz, respectively. Leg stiffness, k_leg_, was calculated by the ratio ΔF/Δ*L* within each phase*,* where ΔF is the change in the resultant ground reaction force, while Δ*L* represents the within-phase change in normalised leg length (by standing leg length) of a 3D vector: from pelvis segment centre of mass to centre of pressure. Given the multiplane nature of limb movements, the multiplanar method is most complete to compute leg stiffness^68^. Gait events were defined using the vertical component of the ground reaction force—an ascending and descending threshold of 20 N identified foot contact (FC), and toe-off (TO), respectively. Within this time period, four other events were created from the body-weight normalised ground reaction force signal when it exceeded both 0.2 and 1.0 body weight (BW), when it reached a maximum, and when it descended below 0.2 BW. The stance period was sub-divided into three task-relevant sub-phases: touch-down (K1, from 0.2 to 1BW); loading (K2, from 1BW to peak force); and unloading (K3, from peak force to 0.2BW). A stride-by-stride time series of k_leg_ for each of the three phases was exported to Matlab (The MathWorks Inc., Massachusetts, US) for computing stiffness control parameters: mean, coefficient of variability (CV), and statistical persistence (DFAα).

Detrended Fluctuation Analysis (DFAα) was developed to measure the scaling index of complex systems, such as the locomotor control system. For stride-to-stride time-interval regulation, the DFAα reveals long-range correlations, indicating an underlying allometric self-organised control process^38^. However, an alternative perspective of the DFAα is that complexity can be affected by an external agent, such as central nervous system intervention. Here, the DFA was applied to contrast inter-related parameters of gait to reveal goal-relevant parameters that are under tight control^33^. Both empirical data and simulation models of the locomotor control system demonstrate that either a regulating external agent, or reduction of inherent complexity, can have similar effects on the break-down of statistical persistence^37,69,70^.

DFAα values from 0.6 to 1.0 indicate relatively higher statistical persistence; while a break-down of persistence occurs when α values converge towards 0.5^33,71^. Under the model of hierarchical locomotor control, the minimum intervention principle and dynamical systems theory, α values are interpreted as the combined product of both control regulation (cognitive, high-level control) and system complexity (biological, low-level control). Specifically, high α values (≈ 1.0) can be due to either loose control regulation or a highly complex system, while low α values (≈ 0.5) can be due to either tight control regulation or a system that has reduced complexity. The interpretation of results related to DFA require an understanding based on the two-system control hierarchy model previously explained. Under this model, both control levels can independently effect a reduction in statistical persistence; reflecting a constraint of embodied neuro-musculoskeletal entropy at the low level, or increased control regulation from high level (Fig. 5).

It has been shown that signal complexity is reduced in locomotor systems affected by disease and ageing^42^, and from fatigue and injury^46^. Essentially, these biologically affected locomotor systems also demonstrate a loss of persistence; but in contrast to control regulation effects on persistence, the biological effects are indicators of an inherently less complex and flexible system. Two investigations by Dingwell, Bohnsack-McLagan^44^ and Dingwell and Cusumano^33^ used experimental data to validate the theory that persistence is an indicator of central nervous system intervention to correct goal-relevant deviations of gait parameters. In the present study we adopt this signal analysis tool and general control regulation theory—but without the model validation—and employ it to assess empirical data of stride-to-stride leg stiffness time-series.

A system with an expanded level of entropy will express persistence in time-series and its processes will functionally interact within and between spatio-temporal scales^72^. Such a flexible system will have a larger set of abundant solutions to satisfy the goals (length–force dynamics) of the control system^73^. There is more likelihood that the high entropy system will self-regulate divergent trajectories to a stable state through its inherent allometric control processes^74^. This suggests that an optimal leg length–force (leg stiffness) state can emerge as a goal-relevant solution from a low-level control process. Therefore, in a high entropy system, there will be less need for intervention on divergent trajectories, and such parameters represented as a time series will show relatively high statistical persistence (approximating 1/f-type noise). In essence, the low-level allometric control processes of a high entropy system are highly flexible. We computed statistical persistence from a customised Matlab program that followed conventional DFA methods^38,75^. Specifically, the scaling exponent was derived from average fluctuations computed from a linear line-of-best-fit for non-overlapping equal sized windows (time scales) of length, w = [9, 17, 33, 65, 129].

To test the hypothesis that a high-level control intervention will determine a reduction in k_leg_ variability, we compared the between-trial change in control process (ΔDFAα) and change in performance outcome (ΔCV) and analysed whether control process is related to performance outcome. For minimal-assisted forefoot loading runners (FFS), changes in DFAα and CV were computed between running trials in moderately assisted shoes (med MI) and minimal assisted shoes (high MI). Likewise, for shoe-assisted rearfoot loading runners (RFS), changes were computed between running trials in high assistance shoes (low MI) and in moderate assisted shoes (med MI). Results were also combined for RFS and FFS within each task-dependent phase of stance (K1, K2, and K3). Moreover, to confirm a change in mean k_leg_ variability, we quantified the distribution of force-leg length values at the final state of K1 and K2 by fitting a 2D ellipse with 95% confidence; the ellipse area in K2 was first normalised by the CV in K1, then the change in ellipse area was computed following the same logic explained above for DFAα and CV.

### Statistical analysis

All data were normally distributed on the basis of the Shapiro-Wilks test. To test contributions of low-level and high-level control, Pearson *r* correlations were performed between change in control process (ΔDFAα) and change in performance outcome (ΔCV) at K1, K2 and K3; likewise, change in distribution (ΔEllipse Area) of mean k_leg_ values were correlated to change in DFAα and CV at K1, K2 and K3. Mean and coefficient of variation (CV) were computed for each *group* × *shoe* × *phase* condition. Because the biomechanical attributes and functional roles between left and right limbs can often be asymmetric, we considered dominant and non-dominant limbs of the participants as separate cases (i.e. n_FF_ = 20, n_RF_ = 20). A mixed design 3-factor (*group* × *shoe* × *phase*) repeated-measures ANOVA was used to examine the interaction and main effects of within-subject factors of *shoe* (3 levels: low MI, medium MI, high MI) and task-dependent *Phase* (3 levels: K1, K2 and K3—touch-down, loading, unloading), and between-subject factor of foot loading type *Group* (2 levels: forefoot, rearfoot) on the three dependent variables of k_leg_ (mean) and k_leg_ control (CV, DFAα). Significance was set at 0.05 for all tests. Planned contrasts examined specific levels of an interaction effect between *group, phase and shoe*. Tukey post-hoc analysis was used to test multiple pairwise comparisons. All statistics were performed using SPSS software (version 25, SPSS Inc., Chicago, IL, USA).

## Supplementary Information

Supplementary Tables.

## Data Availability

The datasets generated during and/or analysed during the current study are available from the corresponding author on reasonable request.
