# Supplementary material for: Insight into the hierarchical control governing leg stiffness during the stance phase of running
Source: Sci Rep. 2022 Jul 15;12:12123. doi: 10.1038/s41598-022-16263-7 (PMC9287449; doi:10.1038/s41598-022-16263-7)
Supplement: Supplementary file 1 — Supplementary Tables. [file 41598_2022_16263_MOESM1_ESM.pdf]

Insight into the Hierarchical control governing leg stiffness during the stance phase of running

Alessandro Garofolini, Karen J Mickle, Patrick McLaughlin, and Simon B Taylor

Appendixes

Table S1. Group mean and CV for leg stiffness and alpha level in the three functional phases of touchdown (K1), loading (K2), and unloading (K3). Comparisons are made among the three shoe type (LOW, MED, HIGH).

	LOW		MED		HIGH		POOLED	
	**FFS**	**RFS**	**FFS**	**RFS**	**FFS**	**RFS**	**FFS**	**RFS**
**K1**								
mean	66.95 (35.34)	56.78 (18.40)	71.15 (33.70)	80.56 (25.82)	76.96 (55.30)	137.43 (55.26)	71.69 (42.07)	91.59 (49.72)
cv	27.6 (6.21)	19.04 (5.13)	38.92 (19.11)	24.06 (6.48)	36.63 (29.33)	31.97 (14.51)	34.38 (20.77)	25.02 (10.89)
alpha	0.72 (0.13)	0.61 (0.11)	0.68 (0.13)	0.60 (0.11)	0.65 (0.11)	0.62 (0.15)	0.68 (0.13)	0.61 (0.12)
**K2**								
mean	27.23 (6.45)	27.86 (5.04)	26.46 (5.81)	25.89 (4.55)	26.17 (7.43)	23.29 (5.04)	26.62 (6.50)	25.68 (5.16)
cv	11.80 (3.38)	8.47 (2.05)	14.90 (5.60)	10.41 (2.37)	13.74 (7.46)	18.26 (7.31)	13.48 (5.78)	12.38 (6.21)
alpha	0.64 (0.09)	0.64 (0.09)	0.65 (0.10)	0.61 (0.10)	0.61 (0.09)	0.61 (0.08)	0.63 (0.09)	0.62 (0.09)
**K3**								
mean	16.00 (2.00)	22.37 (6.84)	15.78 (2.05)	16.45 (1.73)	16.32 (1.87)	20.03 (5.33)	16.03 (1.95)	19.62 (5.58)
cv	10.22 (3.82)	7.42 (1.68)	10.69 (3.72)	7.2 (1.35)	7.53 (1.55)	12.88 (7.93)	9.48 (3.45)	9.17 (5.36)
alpha	0.75 (0.10)	0.7 (0.18)	0.71 (0.11)	0.67 (0.13)	0.76 (0.10)	0.66 (0.12)	0.74 (0.10)	0.68 (0.14)

Table S2. Shoes characteristics and minimalist index classification.

	**Mizuno Wave Rider 21**	**Mizuno Wave Sonic**	**Vibram Five Fingers**
**Shoe features**			
Stability and Motion control (0-5)	0	3	5
Flexibility			
longitudinal (0-2.5)	1	1.5	2.5
torsional (0-2.5)	0.5	1	2.5
Heel to toe drop (0-5)	1	3	5
Stack height (0-5)	0	2	5
Weight (0-5)	2	3.5	4
**Minimalist index**	**18%**	**56%**	**96%**
